# The Influence of CYP2B6 Variants and Administration of Propofol on Patient Outcomes after Traumatic Brain Injury

**DOI:** 10.1089/neur.2024.0025

**Published:** 2024-07-16

**Authors:** Katherine O’Meara, Ava M. Puccio, Dianxu Ren, Sandra Deslouches, Ruchira Jha, David O. Okonkwo, Yvette P. Conley

**Affiliations:** ^1^ University of Pittsburgh School of Nursing, Pittsburgh, Pennsylvania, USA.; ^2^ Department of Neurological Surgery, University of Pittsburgh Medical Center, Pittsburgh, Pennsylvania, USA.; ^3^ Department of Neurology, Translational Neurosciences, Neurosurgery, Barrow Neurological Institute, Phoenix, Arizona, USA.; ^4^ Department of Human Genetics, University of Pittsburgh, Pittsburgh, Pennsylvania, USA.

**Keywords:** cytochrome P-450 CYP2B6, sedatives, traumatic brain injury

## Abstract

Management of severe traumatic brain injury (sTBI) typically involves the use of sedation, which inherently results in benefits and risks. The cytochrome P450 enzyme CYP2B6 is involved in the biotransformation of particular drug classes, including many intravenous sedatives. Variants of the *CYP2B6* gene can lead to decreased systemic clearance of some sedatives, including propofol. This study aimed to investigate the relationship of *CYP2B6* gene variation and patient outcomes after TBI while also considering propofol administration.

Patients who sustained a non-penetrating sTBI and admitted to a single-center Level 1 trauma hospital were included in this study (*n* = 440). The *6 functional allele of *CYP2B6* that leads to reduced enzyme expression and activity required genotyping two single nucleotide polymorphisms, rs3745274 and rs2279343. Patient outcomes were evaluated using the Glasgow Outcome Scale (GOS) and Disability Rating Scale (DRS) at 3 and 6 months post-injury. Data on sedative administration were abstracted from medical records.

Individuals homozygous for the alleles coding for the reduced enzyme expression and activity were more likely to have worse outcomes. A relationship between propofol administration and 3-month GOS and 6-month DRS was noted when controlling for *CYP2B6* genotype.

These findings suggest that genetic variation in *CYP2B6* may influence the impact of intravenous sedation on patient outcomes after TBI and warrants further investigation.

## Introduction

Traumatic brain injury (TBI) is a leading cause of death and disability in the United States, accounting for approximately 2.87 million emergency department visits during 2014.^1^

Severe TBI (sTBI) patients require extensive medical management during their acute, intensive care unit (ICU) care to prevent secondary injury to the brain. This management includes sedation to decrease metabolic demands that the injured brain may experience, including to reducing intracranial pressure (ICP), optimizing ventilation, and reducing the patient’s potential stress response.^2,3^ Common sedatives include propofol, benzodiazepines, and opioids.^3^ Propofol has become a first-line sedative for TBI patients in the ICU due to its rapid onset and short duration of action, minimal effect on renal and hepatic systems, and its neuroprotective properties, including the ability to decrease ICP.^4^ Disadvantages of propofol include amnesia at low doses, lack of analgesic effects, tolerance, decreased mean arterial pressure and cerebral perfusion pressure, prolonged sedation, and propofol infusion syndrome that is a rare, potentially fatal drug reaction characterized by severe metabolic acidosis and circulatory collapse.^4,5^ Propofol is principally metabolized in the liver through conjugation and hydroxylation. The cytochrome P450 enzyme CYP2B6 is involved in the biotransformation of some anesthetic agents, including propofol and ketamine.^6,7^

Genetic polymorphisms may influence outcomes after sTBI.^8^ Likewise, propofol metabolism varies in relation to CYP2B6 gene composition. The most common functionally deficient allele for the *CYP2B6* gene is CYP2B6*6 (Q172H, K262R), which occurs at frequencies of 15–60% in different populations.^7^ This allele leads to slower metabolism of propofol due to lower expression in the liver as alternative splicing of mRNA leads to a reduction in the amount and activity of the enzyme.^6,7,9^ Slower metabolism of propofol could lead to systemic build-up, which could place patients at an increased risk of prolonged sedation and developing propofol infusion syndrome.^10,11^

Although variants of the *CYP2B6* gene have been shown to disrupt normal functioning of anesthetic metabolism, and multiple studies have focused on anesthetic use in TBI patients, there has been little research done combining both. In the current study, we examined the relationship between *CYP2B6* variants and patient outcomes after sTBI in the context of sedation management with intravenous propofol administration.

## Methods

### Setting and description of participants

Data and biospecimens were extracted from a prospective database of sTBI patients treated at UPMC Presbyterian Hospital under a protocol approved by the University of Pittsburgh Institutional Review Board. Written informed consent was obtained from a legal authorized representative. If the patient recovered cognitively during follow-up, they were consented for continued participation. Inclusion criteria included a closed head injury with a hospital-admission Glasgow Coma Scale (GCS) score ≤8, aged 16–80 years, and admitted to the ICU for acute management. Patients had placement of an external ventricular drain for ICP monitoring and cerebrospinal fluid (CSF) diversion as a standard of care.

### Medication and outcome data collection

Medications administered during the hospital stay were extracted from electronic health records with use of propofol coded as yes or no. Outcome data collection consisted of a neuropsychological battery that included the Glasgow Outcome Scale (GOS) and Disability Rating Scale (DRS). The GOS-Extended was performed in addition to GOS in later enrollment years; however, to obtain a larger sample set, the GOS score was used for this analysis. Evaluations were conducted at 3 and 6 months post-injury by a trained neuropsychological technician.

The GOS investigates a participant’s ability to function independently and care for themselves. The GOS is rated based on the following scale: 1 = death, 2 = persistent vegetative state, 3 = severe disability, 4 = moderate disability, and 5 = good recovery.^12^

The DRS is used to evaluate participant’s functional outcomes and abilities after a TBI. The participants are rated on a scale of 0–30, where a score of 0 is indicative of no disability, whereas a score of 30 indicates death. There are three primary categories of impairment studied, including impairment, disability, and handicap. There are seven subcategories, including eye-opening, motor responses, communication, cognitive skill necessary for self-care, physical/cognitive abilities, over-all dependence, and employment.^13^

### Genotype data collection

For this study, DNA samples were extracted from one of two sources. The preferred source was venous blood. The blood was centrifuged to isolate the white blood cells, and then DNA was extracted using a salting out protocol.^14^ The secondary source was DNA extracted from CSF drainage into a ventriculostomy bag that would have otherwise been discarded. The DNA from CSF was extracted using the QIAamp Mini Kit (Qiagen, Valencia, CA, USA) following the manufacturer’s protocol.

The CYP2B6*6 allele is identified by genotyping two single nucleotide polymorphisms (SNPs; rs3745274, 516 G > T and rs2279343, 785A>G). Both of these polymorphisms were genotyped using commercially available TaqMan Allele Discrimination Assays (Thermo Fisher Scientific). For quality control, data were double-called by two individuals blinded to study outcomes and non-concordance rectified by re-visiting the raw data or re-genotyping. Negative controls were included, and significant deviation from Hardy–Weinberg Equilibrium was tested.

### Statistical analysis

The independent variables in this study were the *CYP2B6* genotypes and if the patient received propofol or not. Genotypes and propofol administration were also considered covariates and controlled for in some analyses. The dependent variables are GOS and DRS outcomes. Covariates included in all analyses were sex, age, and initial brain injury severity (GCS). Severity of injury GCS scores were dichotomized into severity of injury, with GCS scores of 3–4 (24.4%) being compared with GCS scores of 5–8 (75.6%). GOS was dichotomized into poor outcomes (GOS 1, 2) and favorable outcomes (GOS 3, 4, 5) and logistic regression performed. Each timepoint was analyzed separately. The GOS and DRS were analyzed at 3 months and 6 months using a chi-squared test and then analyzed by both propofol use and genotype using a two-way ANOVA test. Subgroup analyses were performed for sex and age (<21 or 21 years and older). The age of 21 was used because protocols for propofol use to reduce propofol infusion syndrome in this clinical setting differ for those less than 21. Findings for this exploratory study were considered significant if the *p* value was ≤0.05. Confidence intervals (CIs; 95%) and odds ratios (ORs) were also calculated.

## Results

The final sample set analyzed comprised 440 sTBI participants with complete clinical and genotype data. The average age was 37.5 years (range 16–77; mean and SD 37.51 ± 16.78), with the population being mostly White (*n* = 407) males (*n* = 347) with 80.9% receiving propofol (*n* = 356). The most common rs2279343 genotype being AA (*n* = 188) and most common rs3745274 genotype being GG (*n* = 210). Both SNPs (rs2279343 and rs3745274) did not deviate significantly for Hardy–Weinberg equilibrium. The most common sedatives used in these patients were morphine (24%), fentanyl (56%), and propofol (88%) with other sedatives at less than 1%, and all were administered continuously with dosage and duration varying based on the patient’s level of sedation. Propofol was administered alone 27% of the time, fentanyl and morphine alone 2% and 3% of the time, respectively, with the most common combination being propofol and fentanyl (41%) followed by propofol and fentanyl and morphine (12%), propofol and morphine (8%), and fentanyl and morphine (<1%). Demographic data for this study are shown in Table 1.

**Table 1.** Study Demographics

Characteristics	TBI (*n* = 440)
Age (years; mean ± SD)	37.51 ± 16.78
Sex	
Male	347 (78.86%)
Female	93 (21.14%)
Race	
White	407 (92.5%)
Asian, Indian	3 (0.68%)
Black, African American	29 (6.59%)
Other	1 (0.23%)
Glasgow Coma Scale	
3–4	107 (24.43%)
5–8	331 (75.57%)

After analysis using an Analysis of Maximum Likelihood Estimates, when controlling for *CYP2B6* genotype, propofol was found to be significant for GOS at the 3-month timepoint (*p* = 0.0432). It was also found that the rs3745274 genotype GT is significant for more favorable outcomes than GG for GOS at 6 months (*p* = 0.0451) (Table 2).

**Table 2.** Analysis of Maximum Likelihood Estimates for GOS for rs2279343 and rs3745274

SNP and GOS	Estimate	Standard error	Wald Chi-Square	*p* value
rs2279343; 3-month GOS				
AA	−1.4359	0.7708	3.4697	0.0625
GA	−1.3095	0.7764	2.8445	0.0917
Propofol; controlling for rs2279343 at 3-month GOS	−0.5588	0.3758	2.2104	0.1371
rs2279343; 6-month GOS				
AA	−0.8891	0.5582	2.5372	0.1112
GA	−1.0117	0.5640	3.2180	0.0728
Propofol; controlling for rs2279343 at 6-month GOS	−0.2421	0.3313	0.5340	0.4649
rs3745274; 3-month GOS				
GG	−0.8310	0.5297	2.4615	0.1167
GT	−0.6052	0.5454	1.2313	0.2672
Propofol; controlling for rs3745274 at 3-month GOS	−0.7744	0.3830	4.0895	0.0432
rs3745274; 6-month GOS				
GG	−0.9401	0.4856	3.7480	0.0529
GT	−1.0003	0.4991	4.0160	0.0451
Propofol; controlling for rs3745274 at 6-month GOS	−0.5550	0.3411	2.6473	0.1037

SNP = single nucleotide polymorphism; GOS = Glasgow Outcome Scale.

The same tests were also done for DRS at 3-month and 6-month timepoints. These findings also align with GOS score, with propofol significantly associated with better 6-month DRS outcomes (*p* = 0.0257) when controlling for *CYP2B6* genotype (Table 3). Subgroup analyses indicate that the impact of propofol for 6-month DRS is only significant (*p* = 0.015; OR: 0.155; 95% CI: 0.034–0.695) in the less than 21-year-old sTBI individuals.

**Table 3.** Analysis of Maximum Likelihood Estimates for DRS for rs2279343 and rs3745274

SNP and DRS	Estimate	Standard error	Wald Chi-Square	*p* value
rs2279343; 3-month DRS				
AA	−0.4373	0.4713	0.8611	0.3534
GA	−0.4315	0.4767	0.8191	0.3654
Propofol; controlling for rs2279343 at 3-month GOS	−0.3060	0.3086	0.9827	0.3215
rs2279343; 6-month DRS				
AA	−0.1171	0.4456	0.0691	0.7927
GA	−0.2040	0.4531	0.2028	0.6524
Propofol; controlling for rs2279343 at 6-month GOS	−0.4539	0.3049	2.2168	0.1365
rs3745274; 3-month DRS				
GG	−0.1041	0.4122	0.0638	0.8007
GT	−0.0750	0.4233	0.0314	0.8594
Propofol; controlling for rs3745274 at 3-month GOS	−0.5905	0.3055	3.7375	0.0532
rs3745274; 6-month DRS				
GG	0.0597	0.4052	0.0217	0.8829
GT	0.0627	0.4190	0.0224	0.8811
Propofol; controlling for rs3745274 at 6-month DRS	−0.6777	0.3038	4.9747	0.0257

SNP = single nucleotide polymorphism; DRS = Disability Rating Scale.

## Discussion

The aim of this study was to investigate the relationship of *CYP2B6* variants on the outcomes of sTBI patients while considering propofol administration. *CYP2B6* codes for an enzyme that is heavily involved in drug metabolism, especially the metabolism of propofol and other frequently used anesthetics. Variants of this gene can cause adverse drug reactions, although little research has been done regarding the impact this gene may have on sTBI patients, in whom an adverse drug reaction could be potentially life-threatening.

One of the most studied *CYP2B6* variants is the CYP2B6*6, which is a combination of CYB2B6*4 and CYP2B6*9 alleles. The rs3745274 (determines the *9 allele) and rs2279343 (determines the *4 allele) SNPs are used to assign the CYP2B6*6 allele. The *6 allele is known to result in decreased protein function and expression, and the frequency of this allele differs greatly across populations.^15^ Phenotypically, for rs3745272 and rs2279342, respectively, GG and AA homozygotes are rapid metabolizers, GT and AG heterozygotes have intermediate activity of the enzyme, and TT and GG homozygotes are slow metabolizers.^16^ For pharmacologic considerations, GG and AA homozygotes should be given an increased dose of the drug, GT and AG heterozygotes should be given a traditional therapeutic dose of the drug, and TT and GG (i.e., *6) homozygotes should be given a decreased dose.^16^

No significant interaction between genotype and propofol on patient outcomes after sTBI was found; however, when controlling for propofol, *6 homozygotes had worse outcomes at 3-month GOS. The *6 homozygotes phenotypically are slow metabolizers indicating that patients with this genotype can achieve therapeutic benefits with reduced doses and avoid adverse events. For sTBI patients in the ICU, genotype for *CYP2B6* could impact drug metabolism and systemic clearance that then impacts sedation and ICP levels, which can then impact recovery, potentially explaining the relationship between *CYP2B6* genotype and patient outcomes. In sTBI patients, increased sedation may also increase the risk of delirium and cognitive dysfunction. Furthermore, the slow metabolization of propofol caused by this variant may increase risk for propofol infusion syndrome. While collecting data, there were no noted instances where propofol infusion syndrome occurred, but there were many instances where the dose or choice of sedative was changed during the patient’s ICU stay to achieve optimized sedation, whether that be the patient being too sedated or not sedated enough. The most common sedatives used in these patients were morphine, fentanyl, and propofol. In addition, standing orders for propofol use include checking serum triglyceride levels 48 h after admission if the patient is receiving propofol, then daily if the patient is receiving a propofol infusion greater than 60 mcg/kg/min for more than 24 h, or if the patient is less than 21 years old. Triglyceride levels are also monitored, and if abnormally high, propofol will be discontinued. It is also interesting to note the impact of the *6 variant on 3-month GOS was not found for 6-month GOS, which may indicate that long-term effects may be less of an issue. Once the patient leaves the hospital or rehabilitation, the type and dosage of the drug administered are re-examined in a rehabilitation setting.

When controlling for the genotype, propofol is significantly associated with worse 3-month GOS and 6-month DRS. This means that after receiving the drug, patients are more likely to have decreased independence, ability to perform activities of daily living, as well as decreased communication abilities. This could be due to potential adverse drug reactions. Studies have shown that using propofol at a higher dose than recommended or for a long period of time could lead to increased cognitive impairment, as well as other adverse effects such as cardiac, kidney, liver, and pancreatic dysfunction.^17^ Having a neurological insult adds to the potential for this to happen. If these events do happen, this can greatly increase their length of stay in the hospital and additional treatments, which increases risk for complications. This remains independent of patient’s genotypes, showing that this relationship is most likely due to drug dosage and time given. Interestingly, through subgroup analyses, being under age 21 increased the relationship between propofol and 6-month DRS. This relationship can be explained as adverse effects of propofol are shown to be more common in younger people.^4^

Limitations of this study include the retrospective design and charting systems, not accounting for therapeutic intensity or all medications, heterogeneity of mechanism of injury, polysubstance use, and statistical use of multiple testing. The data collected on drugs used were collected for the period from 2000 to 2016. Some participants were excluded due to the lack of these data being complete for the study purposes, which may have biased our results. sTBI is heterogeneous in presentation, and intracranial finding patterns and patients have different comorbidities that could potentially compromise their recovery, which were not accounted for in these analyses. This study also only focused on the inclusion of propofol—if the patient received it at any time throughout their stay or not—but many of the patients received other sedatives, diuretics, antiepileptics, and other medications throughout their stay. This polysubstance use could also impact the metabolism of propofol, as well as the chances of adverse reactions and complications that the patient could experience. There could also be a combined effect that could impact the patient’s recovery both positively and negatively. Another limitation was the statistical multiple tests performed and lack of diversity among the participants, limiting the generalizability of our pilot findings and the need to perform this study in a larger and more diverse patient population.

Because this study only focused on the inclusion of propofol, future research in this area could investigate additional therapeutics, as well as the difference in outcomes when receiving high-dose propofol versus low-dose propofol, as well as the time period the patient receives propofol. This research could provide more insights on the recommended propofol dosages based on genotype.

These findings are indicative that personalized medicine approaches to treating patients with a sTBI need to be considered and that genetic polymorphisms are impactful and may affect patient outcomes. More research about medication dosages needs to be conducted, but this serves as a basis to show that genotyping patients to tailor medication dosages can potentially promote better patient outcomes and recovery.

## Authorship Confirmation/Contribution Statement

K.O.: conceptualization, data collection, and writing first draft; A.P.: conceptualization, data collection, and editing and approving final draft; D.R.: data analyses and editing and approving final draft; S.D.: data collection and editing and approving final draft; R.J.: conceptualization and editing and approving final draft; D.O.: data collection and editing and approving final draft; Y.C.: conceptualization, data collection, writing first draft, and editing and approving final draft.

## Abbreviations Used

CSF: cerebrospinal fluid
CYP2B6: cytochrome P450 2B6
DNA: deoxyribonucleic acid
DRS: Disability Rating Scale
GCS: Glasgow Coma Scale
GOS: Glasgow Outcome Scale
ICP: intracranial pressure
ICU: intensive care unit
NIH: National Institutes of Health
SD: standard deviation
sTBI: severe traumatic brain injury
TBI: traumatic brain injury
